# Impact of metabolic syndrome and its components on cardiovascular disease event rates in 4900 patients with type 2 diabetes assigned to placebo in the field randomised trial

**DOI:** 10.1186/1475-2840-10-102

**Published:** 2011-11-21

**Authors:** Russell Scott, Mark Donoghoe, Gerald F Watts, Richard O'Brien, Christopher Pardy, Marja-Riitta Taskinen, Timothy ME Davis, Peter G Colman, Patrick Manning, Gregory Fulcher, Anthony C Keech

**Affiliations:** 1 Lipid & Diabetes Research Group, Christchurch Hospital, Christchurch, New Zealand; 2 NHMRC Clinical Trials Centre, University of Sydney, Sydney, Australia; 3 School of Medicine and Pharmacology, University of Western Australia, Perth, Australia; 4 School of Medicine, University of Melbourne, Melbourne, Australia; 5 Department of Medicine, Helsinki University Central Hospital and Biomedicum, Helsinki, Finland; 6 Department of Medical and Surgical Sciences, Dunedin School of Medicine, Dunedin, New Zealand; 7 Department of Diabetes, Endocrinology and Metabolism, Royal North Shore Hospital, Sydney, Australia

## Abstract

**Background:**

Patients with the metabolic syndrome are more likely to develop type 2 diabetes and may have an increased risk of cardiovascular disease (CVD) events.We aimed to establish whether CVD event rates were influenced by the metabolic syndrome as defined by the World Health Organisation (WHO), the National Cholesterol Education Program (NCEP) Adult Treatment Panel III (ATP III) and the International Diabetes Federation (IDF) and to determine which component(s) of the metabolic syndrome (MS) conferred the highest cardiovascular risk in in 4900 patients with type 2 diabetes allocated to placebo in the Fenofibrate Intervention and Event Lowering in Diabetes (FIELD) trial.

**Research design and methods:**

We determined the influence of MS variables, as defined by NCEP ATPIII, IDF and WHO, on CVD risk over 5 years, after adjustment for CVD, sex, HbA_1c_, creatinine, and age, and interactions between the MS variables in a Cox proportional-hazards model.

**Results:**

About 80% had hypertension, and about half had other features of the metabolic syndrome (IDF, ATPIII). There was no difference in the prevalence of metabolic syndrome variables between those with and without CVD at study entry. The WHO definition identified those at higher CVD risk across both sexes, all ages, and in those without prior CVD, while the ATPIII definition predicted risk only in those aged over 65 years and in men but not in women. Patients meeting the IDF definition did not have higher risk than those without IDF MS.

CVD risk was strongly influenced by prior CVD, sex, age (particularly in women), baseline HbA1_c_, renal dysfunction, hypertension, and dyslipidemia (low HDL-c, triglycerides > 1.7 mmol/L). The combination of low HDL-c and marked hypertriglyceridemia (> 2.3 mmol/L) increased CVD risk by 41%. Baseline systolic blood pressure increased risk by 16% per 10 mmHg in those with no prior CVD, but had no effect in those with CVD. In those without prior CVD, increasing numbers of metabolic syndrome variables (excluding waist) escalated risk.

**Conclusion:**

Absence of the metabolic syndrome (by the WHO definition) identifies diabetes patients without prior CVD, who have a lower risk of future CVD events. Hypertension and dyslipidemia increase risk.

## Introduction

Patients with the metabolic syndrome (MS) events are also more likely to develop type 2 diabetes and may have an increased risk of future cardiovascular disease (CVD) [1].

The most commonly used guidelines for definitions of MS are those developed by the National Cholesterol Education Program (NCEP) Adult Treatment Panel III (ATPIII) in 2001 [2], the World Health Organization (WHO) in 1999 [3], and more recently, the International Diabetes Federation (IDF) definition [4], and the harmonized definition [5]. The various components of MS do not contribute equally to CVD risk in those without frank diabetes, and the relative importance of clusters of these risk factors in the setting of established diabetes is less clear.

In the Fenofibrate Intervention and Event Lowering in Diabetes (FIELD) study [6,7], 9795 patients with type 2 diabetes, with or without prior CVD, were recruited and followed up for an average of 5 years. Of these, 4900 were randomized to placebo, providing a sample size sufficient to explore whether CVD event rates were influenced by the presence of MS components (hypertension, dyslipidemia, and waist circumference) or clusters, according to the 4 current definitions. This study also allowed us to assess whether various MS components affected the risk of CVD equally or differently in those with and those without prior CVD and in predefined age and sex subgroups.

## Methods

In the FIELD study, 9795 patients with type 2 diabetes, aged 50 to 75 years, from Australia, New Zealand, and Finland were randomly assigned to treatment with placebo or fenofibrate for a planned duration of 5 years. The trial has been described in detail elsewhere [6]. To be eligible, patients had to have an initial plasma total cholesterol level of 3.0-6.5 mmol/L, plus a total cholesterol/HDL-c ratio of ≥ 4.0 or plasma triglyceride 1.0-5·0 mmol/L, with no clear indication for lipid-modifying therapy at study entry. Exclusion criteria included renal impairment (creatinine > 130 μmol/L), chronic liver disease or symptomatic gallbladder disease, and a cardiovascular event within 3 months before recruitment.

In this further study of FIELD participants, we examined whether the presence of MS, and its various components, increased cardiovascular disease events over 5 years. In addition, we examined which components of MS contributed most to cardiovascular risk. This analysis was confined to the placebo group (*n *= 4900, including 1833 women), who did not receive fenofibrate during the trial, and of whom 1016 had known CVD at study entry.

The prevalence at baseline of the MS components for referenced definitions (ATPIII, IDF, and WHO) was determined (Table 1). The harmonized definition data added nothing to the conclusions regarding the IDF definition, so were not considered further. High blood pressure was defined as a self-reported history of hypertension and documented use of medication for hypertension, or a mean of 3 blood pressure values at baseline above 130/85 mm Hg for the IDF, ATPIII and harmonized definitions, or above 140/90 mm Hg for the WHO definition [3-5,8]. In the WHO MS definition, dyslipidemia is a single component, and patients can have either raised blood triglyceride (≥ 1.7 mmol/L) or low HDL-c levels (< 0.9 mmol/L for men and < 1.0 mmol/L for women). If both were present, this was counted as a single risk factor in all analyses using the WHO definition, whereas the other definitions allowed each to be counted individually. Thus, if HDL-c levels were low and triglyceride was high, both risk factors were counted separately for IDF and ATPIII. Type 2 diabetes was a core component for each of the definitions, and waist measurement was included as a requirement of the IDF definition.

**Table 1:** Prevalence of features of metabolic syndrome at baseline in patients assigned to placebo in the FIELD study (points of difference in criteria are shown in bold)

Feature of metabolic syndrome	Men	Women	All patients
	(n = 3067)	(n = 1833)	(n = 4900)
**ATPIII criteria (any 3)**			
Diabetes or impaired fasting glucose	100	100	100
High waist measurement (**M > 102 cm, F > 88 cm**)	54.5	80.6	64.3
Hypertension history or BP ≥ 130/85 mmHg	82.2	85.8	83.6
High triglycerides (≥ 1.7 mmol/L)	50.0	54.1	51.5
Low HDL cholesterol (M < 1.03 mmol/L, F < 1.29 mmol/L)	54.8	66.2	59.1
*Metabolic syndrome according to ATPIII*	78.3	90.3	82.8
**IDF criteria (waist + any 2)**			
Diabetes or impaired fasting glucose	100	100	100
High waist measurement (M ≥ 94 cm, F ≥ 80 cm)	83.9	95.0	88.0
Hypertension history or BP ≥ 130/85 mmHg	82.2	85.8	83.6
High triglycerides (≥ 1.7 mmol/L)	50.0	54.1	51.5
Low HDL cholesterol (M < 1.03 mmol/L, F < 1.29 mmol/L)	54.8	66.2	59.1
*Metabolic syndrome according to IDF*	80.5	92.5	85.0
**Harmonized criteria (any 3)**			
Diabetes or impaired fasting glucose	100	100	100
High waist measurement (M ≥ 94 cm, F ≥ 80 cm)	83.9	95.0	88.0
Hypertension history or BP ≥ 130/85 mmHg	82.2	85.8	83.6
High triglycerides (≥ 1.7 mmol/L)	50.0	54.1	51.5
Low HDL cholesterol (M < 1.03 mmol/L, F < 1.29 mmol/L)	54.8	66.2	59.1
*Metabolic syndrome according to harmonized definition*	87.6	94.7	90.3
**WHO criteria (diabetes + any 2)**			
Diabetes or impaired fasting glucose	100	100	100
**High waist-hip ratio (M > 0.9, F > 0.85) or BMI > 30**	88.5	80.5	85.5
Hypertension history or blood pressure **≥ 140/90 mmHg**	68.5	73.3	70.3
High triglycerides (≥ 1.7 mmol/L) and/or **low HDL-c (M < 0.9 mmol/L, F < 1.0 mmol/L)**	58.9	59.0	58.9
**Microalbuminuria (urine albumin/creatinine ≥ 3.4 mg/mmol)**	23.6	20.8	22.6
*Metabolic syndrome according to WHO*	82.6	80.7	81.9

* IDF criteria for hypertension, high triglyceride, and low HDL-c are the same as those for ATPIII.

† Harmonized criteria are the same as for IDF except metabolic syndrome does not require high waist measurement.

‡ Ethnic and sex-specific cut-offs for waist circumference define high risk in the harmonized definition. This analysis, for a population mainly of European origin, used the "Caucasian" waist cut-off.

FIELD, Fenofibrate Intervention and Event Lowering in Diabetes; ATPIII, Adult Treatment Panel III; M, male; F, female; BP, blood pressure; IDF, International Diabetes Federation; WHO, World Health Organization; HDL-c, high-density lipoprotein cholesterol

Outcome was defined as the first CVD event over the duration of the study (CVD death, nonfatal coronary events, stroke, and coronary and carotid revascularizations).

Event rates for those meeting or not meeting MS criteria were established in subgroups based on the prespecified cut-off age of 65 years, sex, and the presence or absence of prior CVD. The effects of baseline blood pressure, waist circumference, HDL-c, and triglyceride levels on CVD event rates were analysed by quintile.

### Statistical analysis

We established the CVD risk associated with the various MS components in a Cox proportional-hazards model with categorical data from this cohort using the WHO definition, with adjustment for age, sex, prior CVD, hemoglobin (Hb) A_1c_, and creatinine. A separate Cox proportional-hazards model with continuous variables was used to determine the CVD risk associated with gradients in waist-hip ratio, blood pressure, HDL-c, triglyceride concentrations, and urine albumin-creatinine ratio, with the same adjustment variables. In both models, all possible two-way interactions between predictors were considered for inclusion and retained if *P *< 0.05. For predictors interacting with continuous variables, the hazard ratio at the median of each continuous variable is presented. All variables used in the analyses were measured at study entry.

## Results

### Prevalence of the individual metabolic syndrome components in the FIELD cohort

The prevalence rates of MS defined by ATPIII, IDF and WHO 1999 are detailed in Table 1. Most participants met criteria for MS, regardless of definition. Hypertension was common. The WHO definition led to more similar proportions of men and women having MS than the other classifications, which identified higher prevalence rates of the various features in women than men, particularly for waist criteria. There was no difference in the prevalence of MS or its components in those with or without CVD at study entry (data not shown).

The WHO definition was the most discriminatory for prediction of future CVD events, and predicted higher risks across all predefined subgroups (men and women, age under 65 years or 65 and over, and prior CVD or no prior CVD). (Table 2). The presence of MS by the WHO definition conferred significantly higher CVD risk even among patients who had prior CVD at study entry, whereas the IDF and ATPIII definitions did not identify this subgroup as having increased risk. ATP III criteria were limited to identifying higher risk only in men and those over 65 years. Furthermore, patients meeting the IDF MS definition did not have significantly higher CVD risk than patients without IDF MS and none of the prespecified subgroups were associated with increased CVD risk. These conclusions did not change if the study population was confined to the 3837 patients without prior CVD.

**Table 2:** Total CVD event rates (%, 95% CI) by presence of metabolic syndrome with different definitions in patients assigned to placebo in the FIELD study

	ATPIII			IDF			Harmonized			Who		
Group	No MS	MS	Diff (95% CI)	No MS	MS	Diff (95% CI)	No MS	MS	Diff (95% CI)	No MS	MS	Diff (95% CI)
Age ≥ 65	12	19	7 (3-11)‡	15	18	3 (-2-7)	11	18	7 (2-12)†	8	19	11 (8-14)‡
Age < 65	11	12	0 (-3-3)	13	11	-2 (-5-2)	11	12	0 (-3-4)	7	12	5 (3-8) ‡
Male	12	18	6 (3-9)‡	16	17	1 (-2-5)	12	17	5 (2-9)†	9	18	9 (6-12) ‡
Female	9	10	1 (-4-5)	7	10	2 (-2-7)	7	10	2 (-3-8)	5	11	5 (2-8) ‡
Prior CVD	22	26	3 (-4-10)	26	25	-1 (-9-6)	19	26	7 (-2-16)	18	26	8 (0-15)*
No prior CVD	9	11	2 (-0-5)	11	11	-0 (-3-3)	10	11	1 (-2-4)	6	12	6 (4-8) ‡
All patients	11	14	3 (1-5)*	14	14	-0 (-3-3)	11	14	3 (0-6)*	8	15	8 (5-10) ‡

*P < 0.05, †*P *< 0.01, ‡*P *< 0.001 for the absolute risk difference for metabolic syndrome compared with no metabolic syndrome. The larger the difference, the greater the risk discrimination provided by the definition.

CVD, cardiovascular disease; FIELD, Fenofibrate Intervention and Event Lowering in Diabetes; MS, metabolic syndrome; ATPIII, Adult Treatment Panel III; IDF, International Diabetes Federation; WHO, World Health Organization;

### Effect of various metabolic syndrome components on cardiovascular disease risk

The effects of metabolic-syndrome and other variables on CVD events were modelled in a Cox regression analysis (Table 3). Non-MS variables of sex, age, prior CVD, baseline HbA_1c_, and creatinine had major influences on CVD outcomes. The effects of MS variables on CVD events were calculated after adjustment for these variables. While increases in triglyceride (0.5 mmol/L) and waist circumference (10 cm) did not have significant effects, both a decrease of HDL-c and an increase in systolic BP were associated with significant CVD disadvantage.

**Table 3:** Cox regression model* for the effect of continuous variables, including features of the metabolic syndrome as defined by ATPIII, on the risk of total CVD events in patients assigned to placebo in the FIELD study

Variable†	Hazard ratio (95% CI)	*P*
**Predictive variable**		
Female (at 62 years)	0.70 (0.55-0.88)	0.003
Age (per 10 years): male	1.21 (1.06-1.39)	< 0.001
Age (per 10 years): female	1.74 (1.38-2.19)	
Prior CVD (at 140 mmHg SBP, 6.85% HbA_1c_)	2.14 (1.81-2.53)	< 0.001
Hemoglobin A_1c _(per 1%): no prior CVD	1.18 (1.10-1.26)	< 0.001
Hemoglobin A_1c _(per 1%): prior CVD	1.03 (0.95-1.13)	
Creatinine (per 20 μmol/L)	1.21 (1.09-1.35)	< 0.001
**Metabolic syndrome variable‡**		
Waist -hip ratio (per 0.1)	1.03 (0.91-1.17)	0.60
Systolic BP (per 10 mmHg): no prior CVD	1.16 (1.09-1.24)	< 0.001
Systolic BP (per 10 mmHg): prior CVD	1.01 (0.94-1.09)	
Triglycerides (per 0.5 mmol/L)	1.03 (0.99-1.07)	0.19
HDL-c (per 0.1 mmol/L)	0.94 (0.90-0.97)	< 0.001
Urine albumin-creatinine ratio (per doubling)	1.06 (1.02 - 1.10)	0.002

* Cox proportional-hazards assumptions were met.

† All variables were centered at medians. Standard deviations for distributions of the continuous variables were: age, 6.9 years; HbA_1c_, 1.35%; creatinine, 15.8 μmol/L; waist, 13 cm; systolic BP, 15 mmHg; triglycerides, 0.88 mmol/L; HDL-c, 0.26 mmol/L.

‡ Corrected for age, sex, prior CVD, baseline HbA_1c _and creatinine.

ATPIII, Adult Treatment Panel III; CVD, cardiovascular disease; FIELD, Fenofibrate Intervention and Event Lowering in Diabetes; BP, blood pressure; HDL-c, high-density lipoprotein cholesterol

**Figure 1:**
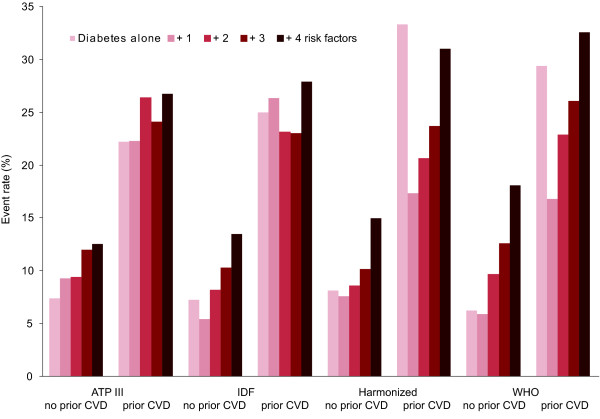
**Cardiovascular disease event rates according to the number of additional metabolic syndrome components (risk factors) at baseline in relation to the ATPIII, IDF, harmonized, and WHO categories in patients allocated to placebo without (*n *= 3837) or with (*n *= 1063) prior cardiovascular disease**. Apparent high event rates in the groups with no additional risk factors by the harmonized and WHO definitions are an artifact of low patient numbers.

Hypertension in patients without prior CVD more than doubled CVD risk in a categorical Cox regression model (data not shown). In those without prior CVD, increasing numbers of MS variables escalated risk regardless of the MS definition being applied (Figure 1); those with diabetes plus all MS components had two to three times the risk of those with diabetes alone (regardless of definition).

The CVD risk of the 1063 patients with prior CVD was 25-26% if they met criteria for MS (any of the definitions; Table 2). Prior CVD roughly tripled the risk of future events in this group compared with those without prior CVD, even when up to two MS risk factors were present. Increasing numbers of MS variables in those with prior CVD did not so clearly increase risk (Table 2, Figure 1).

### MS variables by quintile and CVD events

**Figure 2:**
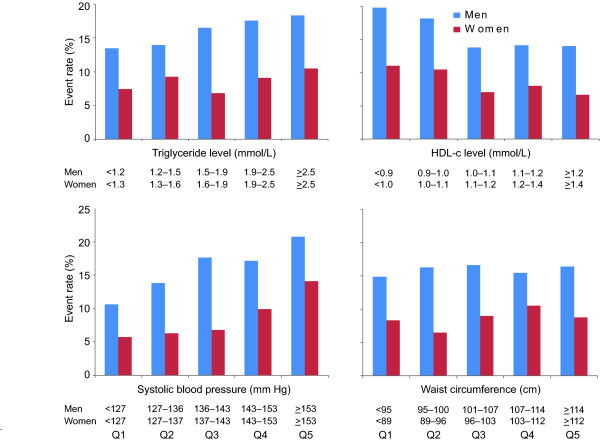
**Cardiovascular disease event rates according to quintiles of baseline triglycerides, HDL-c, systolic blood pressure, and waist circumference in men and women allocated to placebo**.

Men had a higher CVD risk if their HDL-c was below 0.95 mmol/L. No strong gradient of risk was observed for women, although those with HDL-c < 0.98 mmol/L had higher event rates than those with HDL-c > 1.41 mmol/L (Figure 2). For men, a progressive rise in risk occurred as triglyceride levels rose through the quintiles. For women, there was no significant risk gradient. Baseline systolic BP was associated with CVD risk in both sexes across quintiles, but waist measurements were not significantly associated with risk.

## Discussion

In the placebo cohort of FIELD, the absolute risk of a CVD event was higher in those with more MS components, provided they did not have prior CVD. Patients with prior CVD had a less clear risk gradient related to the presence or absence of MS features.

The WHO definition of metabolic syndrome outperformed the other definitions in predicting CVD. It does not include waist measurement, which was not a significant risk factor in any case (replaced by waist-hip ratio and also not a significant risk factor). Its hypertension cut-off is higher than that of the other definitions, and it combines the triglyceride criterion (not predictive in women) with the lower and sex-specific HDL-c criterion. Our findings are in line with those of the Strong Heart Study, also in diabetes, with the WHO definition performing best[9].

The prevalence of MS features, as defined by ATPIII, IDF, and WHO classifications, was high, particularly for treated hypertension or high blood pressure at study entry. Over 80% met MS criteria irrespective of definition, and 60% had at least 3 MS components, in addition to type 2 diabetes.

The protective effect of sex was strong, with women having a 30% lesser CVD risk than men, after adjustment. This is consistent with previous reports, first, that type 2 diabetes doubles in men and triples in women the relative risk of various CVD events [10,11], and second, that men with MS are more likely to have a CVD event than women [12,13]. However, there are also some reports that women are at higher risk of CVD than men [14,15]. In FIELD, for every 10 years of age, at baseline, CVD risk was 74% higher in women and 21% higher in men. Although truncal obesity was prevalent, particularly in women, high waist circumference and waist-hip ratio were not significant CVD risk factors, in contrast to other studies in more general populations [16,17]. This may reflect the greater importance of this component in a prediabetes setting. Although truncal fat is an important driver of insulin resistance [18], and adjustment for WHR has been shown, for example, to influence the relationship of HOMA-index with left ventricular mass in people without diabetes [19], once diabetes is present, it is possible that excess truncal fat no longer predicts future CVD events. However, fatness, adipocytokine release and inflammation are tightly interlinked, and an effect on CVD event rates would have been predicted [20,21]. The most important MS predictor of CVD events in the Framingham Offspring Study was blood pressure [13], followed by HDL-c level, and not waist measurement, consistent with the findings of this FIELD analysis. Waist circumference is a required component of the IDF definition, which may explain its lesser discrimination of risk in comparison with the harmonized version. Of interest, another group has reported an association between prevalence of MS and higher levels of within-normal-limits liver function [22].

The presence of dyslipidemia contributed to CVD risk. All MS definitions apply sex-specific levels for HDL-c, with the triglyceride level set at 1.7 mmol/L, above which LDL particles tend to be of smaller size and greater density, properties that reflect higher atherogenicity [23]. In the FIELD cohort, there was a gradient of risk as triglyceride levels increased above 1.5 mmol/L in men, but in women no change in risk was observed across the quintiles except for a suggestion of higher risk at levels over 2.5 mmol/L. These data support the possibility of a triglyceride risk differential by sex in diabetes, although this was not the finding of the recent meta-analysis of the Emerging Risk Factors Collaboration [24]. It would appear that there is also a strong sex difference with respect to cut-offs for the HDL-c levels that escalate risk. Higher CVD risk in men was associated with HDL-c levels below 0.95 mmol/L, a value similar to that used in these definitions of MS. In women, the FIELD data suggest that the defined levels for HDL-c should be set lower than specified by ATPIII (for example, < 1.0 mmol/L) to select diabetes patients with higher CVD risk. This may partly explain why the WHO definition came out as the most predictive (although it also captured as having MS those with higher blood pressure and microalbuminuria). Thus, the most aggressive risk factor management for diabetes patients might be guided by levels of HDL-c and triglyceride that are sex-specific. For both men and women, an HDL-c level below 1.0 mmol/L seems to best identify highest risk. For triglycerides, a level above 1.5 mmol/L seems appropriate for men, but for women, a much higher value, such as > 2.5 mmol/L, might be necessary to identify higher risk.

The limitations to the study are that the analysis was based on data at study entry, and the proportion of subjects lacking MS features was relatively small. Comparisons between those with and without MS thus might have over- (or under-) estimated effects, but is consistent with other reports[25]. In addition, the average rate of uptake of statins in the placebo group during follow-up was substantial (17% over 5 years) [26], and those with more marked dyslipidemia were most likely to have had this treatment initiated (21%).

## Conclusion

An increasing number of MS variables increased future CVD risk, and for all definitions, other than the IDF definition, the risk of CVD among patients defined as having MS was significantly higher than among those without. The two MS variables that contributed most to this risk were HDL-c and hypertension. Waist measurement was not a material contributor. Of current definitions of MS, the WHO definition, which includes albuminuria, was consistently the best discriminator of risk, whereas the IDF definition was not significantly predictive. These results can help guide clinicians in identifying patients with diabetes at high CVD risk.

## Abbreviations

ATP III: Adult Treatment Panel III; CVD: cardiovascular disease; FIELD: Fenofibrate Intervention and Event Lowering in Diabetes; HDL: high-density lipoprotein; IDF: International Diabetes Federation; LDL: low-density lipoprotein; MS: metabolic syndrome; NCEP: National Cholesterol Education Program; WHO: World Health Organisation.

## Authors' interests

The authors declare that they have no competing interests.

## Authors' contributions

RS designed this study, wrote the manuscript, MD contributed to the discussion, analysed the data, revised the manuscript; GFW contributed to the discussion, revised the manuscript; ROB contributed to the discussion, revised the manuscript; CP contributed to the discussion, analysed the data, revised the manuscript; MRT contributed to the discussion, revised the manuscript; TMED contributed to the discussion, revised the manuscript; PGC contributed to the discussion, revised the manuscript; PM contributed to the discussion, revised the manuscript; GF contributed to the discussion, revised the manuscript; ACK designed the study, contributed to the discussion, wrote the manuscript; all authors read and approved the final manuscript.
